# Identification of housekeeping genes for microRNA expression analysis in kidney tissues of *Pkd1* deficient mouse models

**DOI:** 10.1038/s41598-019-57112-4

**Published:** 2020-01-14

**Authors:** J. J. Muñoz, A. C. Anauate, A. G. Amaral, F. M. Ferreira, R. Meca, M. S. Ormanji, M. A. Boim, L. F. Onuchic, I. P. Heilberg

**Affiliations:** 1 Nephrology Division, Department of Medicine, Universidade Federal de São Paulo, São Paulo, Brazil; 2 Divisions of Molecular Medicine and Nephrology, University of São Paulo School of Medicine, São Paulo, Brazil; 3 Laboratory of Immunology, Heart Institute, University of São Paulo School of Medicine, São Paulo, Brazil

**Keywords:** Gene expression, miRNAs, Polycystic kidney disease

## Abstract

Polycystic kidney disease is a complex clinical entity which comprises a group of genetic diseases that leads to renal cyst development. We evaluated the most suitable housekeeping genes for microRNA expression by RT-qPCR analyses of kidney tissues in *Pkd1*-deficient mouse models from a panel of five candidates genes (*miR-20a*, *miR-*25, *miR-26a*, *miR-191* and *U6*) and 3 target genes (*miR-17*, *miR-21* and *let-7a*) using samples from kidneys of cystic mice (*Pkd1*^flox/flox^:*Nestin*^cre^, CY), non-cystic controls (*Pkd1*^flox/flox^, NC), *Pkd1*-haploinsufficient (*Pkd1*^+/−^, HT), wild-type controls (*Pkd1*^+/+^, WT), severely cystic mice (*Pkd1*^V/V^, SC), wild-type controls (CO). The stability of the candidate genes was investigated using NormFinder, GeNorm, BestKeeper, DataAssist, and RefFinder software packages and the comparative ΔCt method. The analyses identified *miR-26a* as the most stable housekeeping gene for all kidney samples, *miR-20a* for CY and NC, *miR-20a* and *miR-26a* for HT and WT, and *miR-*25 and *miR-26a* for SC and CO. Expression of *miR-21* was upregulated in SC compared to CO and trends of *miR-21* upregulation and *let-7a* downregulation in CY and HT compared to its control kidneys, when normalized by different combinations of *miR-20a*, *miR-*25 and *miR-26a*. Our findings established *miR-20a*, *miR-*25, and *miR-26a* as the best housekeeping genes for miRNA expression analyses by RT-qPCR in kidney tissues of *Pkd1*-deficient mouse models.

## Introduction

Autosomal dominant polycystic kidney disease (ADPKD) is the most frequent monogenic renal disorder, characterized by bilateral development of multiple cysts formation and gradual enlargement of the kidneys^1^. This disease is the fourth leading cause of kidney failure and affects about 1/400 to 1/1000 people worldwide^1^. The genetic and non-genetic factors determines the varying rates at which ADPKD progresses to end-stage renal disease^2^. ADPKD is caused by mutations in *PKD1* or *PKD2*, genes that encode polycystin-1 (PC1) and polycystin-2 (PC2), respectively. *PKD1* mutations are responsible for ~78% of the affected families and are associated with higher disease severity compared to mutations in *PKD2*^3^. PC1 regulates several aspects of tissue morphogenesis and function, which when disturbed promote abnormal cell proliferation, cyst development and transepithelial fluid secretion^2,4–7^.

Several studies have shown that microRNAs (miRNAs) are involved in the pathogenesis of ADPKD^8–10^. Importantly, miRNAs have been proposed as an acquired factor for the disease establishment and responsible for reducing or increasing cyst formation in ADPKD by regulating the expression of multiple target genes associated with cytogenesis^11–19^. A common set of miRNAs is aberrantly expressed in several murine models of polycystic kidney disease (PKD). Three miRNAs have their expression particularly altered in this setting; *let-7a* is downregulated while *miR-17* and *miR-21* are upregulated in kidney cysts and have been associated with PKD progression in mouse models^20–24^.

The heterozygous mouse (HT) is a model heterozygous for a null mutation in *Pkd1*, therefore represented by the genotype *Pkd1*^+/−^. This model reproduces the systemic *Pkd1*-haploinsufficient background found in ADPKD1 patients but virtually with no renal cysts by 15 weeks (wk) of life. Wild-type littermates (*Pkd1*^+/+^; WT) were used as their controls^25,26^. Homozygous mice for a *Pkd1*-floxed allele, in turn, with a mosaic pattern of gene inactivation driven by a Nestin-Cre transgene with consequent excision of exons 2–4 (*Pkd1*^flox/flox^:*Nestin*^cre^; CY), develop a renal cystic phenotype^25,27,28^. The *Pkd1*^flox/flox^:*Nestin*^cre^ mice present cystic kidneys, reproducing the ADPKD phenotype, however do not have a systemic *Pkd1*-haploinsufficiency background. Noncystic animals (*Pkd1*^flox/flox^; NC) were their controls^25,27–29^. The third model comprised a homozygous mouse for a *Pkd1*-knockin hypomorphic allele that prevents PC1 cleavage at the GPS site (*Pkd1*^V/V^; SC). This mouse develops early-onset, post-natal, massive renal cystic disease, characterized by distal nephron involvement, uremia and early mortality^30^. Age-matched wild-type littermates (*Pkd1*^+/+^; WT) were used as their controls.

All three mouse models were maintained on the C57BL/6 strain background, an important requirement for the performance of our study. It must be noted that the WT and CO controls harbor the same genotype (*Pkd1*^+/+^) but are analyzed at different ages. NC controls, in turn, homozygous for the *Pkd1*^flox^ allele but without the *Nestin*^cre^ transgene, do not develop renal cysts^25,30^.

The regulatory function of miRNAs in ADPKD supports their use as potential prognostic and predictive biomarkers as well as therapeutic targets. Their identification, however, requires a reliable and quantitative assessment of miRNA expression. Different methodologies can be used to evaluate miRNA expression, but reverse transcription-quantitative polymerase chain reaction (RT-qPCR) remains the gold-standard for specific detection of selected sets of miRNAs^31,32^. A crucial step to ensure accurate and suitable quantification of PCR data is the normalization of expression levels. This normalization aims to differentiate true biological variations, explain the investigated phenotype and identify non-specific experimentally-induced alterations^33^. In fact, factors such as sample collection and preservation, amount of raw material, enzyme efficiency and RNA integrity can artefactually influence expression levels. Currently, the accepted method of miRNA gene expression normalization is the use of internal reference genes (housekeeping genes)^33^. Ideal housekeeping genes should show no (or minimal) expression variation in the sample under investigation in response to experimental treatment/disease condition.

Despite the growing number of studies investigating miRNA expression in animal models and human polycystic kidney disease^20–24,34^, to the best of our knowledge there is no current consensus on housekeeping genes to be employed in PKD/ADPKD kidney tissue samples. This limitation may restrict study comparisons and most importantly, lead to ambiguous data interpretation and misleading biological conclusions. Several miRNAs currently used as housekeeping genes in other diseases settings display unstable expression profiles, a reality that makes them not suitable for RT-qPCR data normalization^35,36^*. U6* expression profiles, therefore, require appropriate assessment in the PKD scenario, since it has been used as a housekeeping gene in research involving PKD samples^37–39^.

The present study aimed to quantitatively evaluate the performance of four candidate miRNA housekeeping genes (*miR-20a*, *miR-*25, *miR-26a* and *miR-191*) and the *U6* small nuclear RNA and identify the most suitable one/sets for miRNA expression normalization by RT-qPCR in kidney tissue of different *Pkd1*-deficient mouse models.

## Results

### Candidate housekeeping miRNAs: quantitative expression profiles

**Figure 1:**
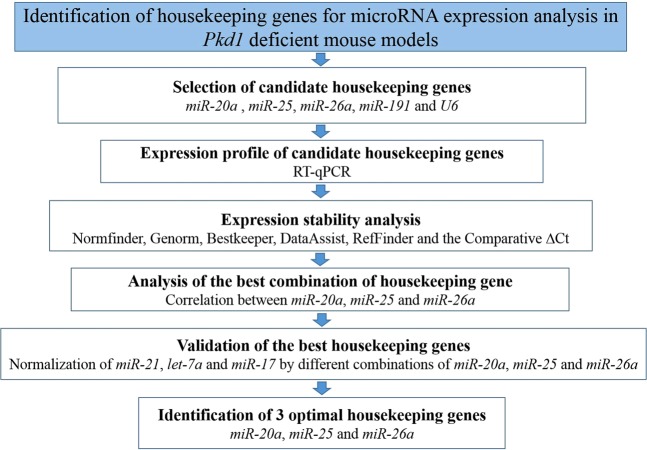
Workflow diagram illustrating strategy for identification of housekeeping normalizer miRNAs for RT-qPCR. *Pkd1*, polycystic kidney disease 1 gene; miR, microRNA. For the sake of comparison, we included housekeeping genes selected from the literature, i.e., *miR-20a*, *miR-25*, *miR-26a*, *miR-191* and *U6*.

Here we applied a stepwise strategy for the identification of the optimal miRNAs as housekeeping genes for miRNA expression by RT-qPCR analyses of kidney tissues in *Pkd1*-deficient mouse models. The scheme workflow is shown in Fig. 1.

The tissue samples were classified into 7 distinct groups: (1) CY, including the cystic kidney samples, n = 10; (2) NC, non-cystic kidney samples, n = 10; (3) HT, *Pkd1*-haploinsuficient kidney samples, n = 6; (4) WT, wild-type kidney samples, n = 6; (5) SC, severely cystic kidney samples, n = 7; (6) CO, early-life, wild-type kidney samples, n = 5; and (7) All, including all kidney samples, n = 44.

**Figure 2:**
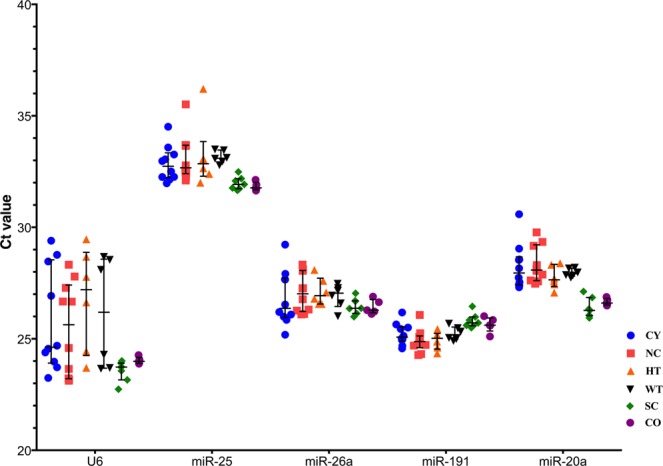
The Ct values of five candidate housekeeping genes. A lower threshold value (Ct) indicates a higher gene expression level. The median values are expressed as horizontal lines, and the error bars represent interquartile range. The Ct values of *miR-25* were the highest, indicating the lowest expression levels.

Before testing the expression stability of the five candidate housekeeping genes, all cDNA samples were normalized at the RNA level. After RT-qPCR cycling, the median Ct values of triplicate reactions were acquired, representing raw expression data. Expression of the *miR-20a*, *miR-*25, *miR-26a*, *miR-191* and *U6* candidate housekeeping genes is shown in Fig. 2.

The median Ct values for *miR-20a* [27.66 (1.26)], *miR-26a* [26.66 (1.07)] and *miR-191* [25.17 (0.70)] lied between 25 and 30, evincing moderate expression. Ct reached its highest value for *miR-25* [32.50 (1.02)], lying above 30, which makes it the lowest expressed gene among the five candidates. Ct values for *U6*, on the other hand, was the lowest one [24.28 (3.95)], placing it as the highest expressed gene (Fig. 2).

Here, we included RT-qPCR analyses of kidney tissues from three mouse models with distinct patterns of *Pkd1* deficiency and correlated them with their respective control tissues. The CY model presented some smaller variations in Ct values compared to its control NC (Fig. 2). *miR-26a* varied by more than 0.5 cycles in CY group but did not exceed 1.0 and Ct varied less than that for the other genes, except for *U6*. The HT model showed higher Ct variability compared to its control WT (Fig. 2). *U6* Ct values varied by more than 1.0 cycle between HT and WT. Expression differences were far lower between SC and its control (CO kidneys); only the *miR-20a* difference exceeded 0.3 cycle.

### Expression stability analysis of the housekeeping miRNAs

The stability values of the candidate housekeeping genes were obtained applying six software packages (Supplementary Table 2). The top-ranked genes (associated with the smallest stability value) are the most stably expressed ones in the set of kidney tissues samples from the three *Pkd1*-deficient mouse models. Apart from *U6* in the HT group, all candidate housekeeping miRNAs presented M values below 1.5, the GeNorm set threshold, findings that are consistent with stability^40^. The Bestkeeper software points out inconsistency when SD is higher than 1.0. The SD value was higher than 1.0 only for *U6* expression in the All, CY, NC, HT and WT sample groups (Supplementary Table 2).

Based on the different utilized algorithms and a visual inspection of all ranks generated by these analyses, *miR-26a* seems to be the best housekeeping gene for the All, HT and CO groups; *miR-20a* for the CY, NC and WT groups; and *miR-25* for the SC group (Table 1).

**Table 1:** Best housekeeping miRNA for each group of samples yielded by software analyses.

Groups	Best housekeeping genes by software						Best housekeeping gene by visual inspection
	NormFinder	GeNorm	RefFinder	ΔCt method	Bestkeeper	DataAssist	
All	*miR-26a*	*miR-26a*	*miR-26a*	*miR-26a*	*miR-191*	*miR-26a*	*miR-26a*
CY	*miR-26a*	*miR-25*	*miR-20a*	*miR-20a*	*miR-191*	*miR-20a*	*miR-20a*
NC	*miR-26a*	*miR-26a*	*miR-20a*	*miR-20a*	*miR-191*	*miR-20a*	*miR-20a*
HT	*miR-26a*	*miR-26a*	*miR-20a*	*miR-20a*	*miR-191*	*miR-26a*	*miR-26a*
WT	*miR-20a*	*miR-25*	*miR-25*	*miR-26a*	*miR-20a*	*miR-20a*	*miR-20a*
SC	*miR-25*	*miR-25*	*miR-191*	*miR-191*	*miR-25*	*miR-25*	*miR-25*
CO	*miR-20a*	*miR-26a*	*miR-26a*	*miR-26a*	*miR-25*	*miR-20a*	*miR-26a*

All, all samples; CY, cystic; NC, non-cystic; HT, haploinsuficient; WT, wild-type; SC, severely cystic phenotype; CO, severely cystic phenotype controls.

NormFinder recommends a SD value lower than 0.5 for genes to be considered relatively stable. Only *miR-20a*, *miR-25* and *miR-26a* had an SD value below 0.5, while *miR-191* and *U6* showed it above 0.5 in the All, CY and NC groups. However, *miR-25* presented SD higher than 0.5 in HT and WT and *miR-191*-related SD lower than 0.5 in SC and CO. While these data represent only a selection of possible tissue pairs (*Pkd1* deficiency kidney tissues versus controls), they illustrate that optimal housekeeping genes can significantly vary between kidneys of mouse models with distinct patterns of *Pkd1* deficiency (Tables 1, 2 and Supplementary Table 2).

**Table 2:** Best combination of housekeeping miRNAs for each group of samples yielded by software analyses.

Groups	Best pair of housekeeping genes by software						Best pair of housekeeping gene by visual inspection	Best trio of housekeeping genes by visual inspection
	NormFinder	GeNorm	RefFinder	ΔCt method	Bestkeeper	DataAssist		
All	*miR-25* + *miR-26a*	*miR-20a* + *miR-26a*	*miR-20a* + *miR-26a*	*miR-20a* + *miR-26a*	*miR-26a* + *miR-191*	*miR-20a* + *miR-26a*	*miR-20a* + *miR-26a*	*miR-20a* + *miR-25* + *miR-26a or miR-20a* + *miR-26a* + *miR-191*
CY	*miR-20a* + *miR-26a*	*miR-20a* + *miR-25*	*miR-20a* + *miR-25*	*miR-20a* + *miR-25*	*miR-25* + *miR-191*	*miR-20a* + *miR-25*	*miR-20a* + *miR-25*	*miR-20a* + *miR-25* + *miR-26a or miR-20a* + *miR-26a* + *miR-191*
NC	*miR-20a* + *miR-26a*	*miR-20a* + *miR-26a*	*miR-20a* + *miR-26a*	*miR-20a* + *miR-26a*	*miR-20a* + *miR-191*	*miR-20a* + *miR-26a*	*miR-20a* + *miR-26a*	*miR-20a* + *miR-26a* + *miR-191*
HT	*miR-20a* + *miR-26a*	*miR-20a* + *miR-26a*	*miR-20a* + *miR-26a*	*miR-20a* + *miR-26a*	*miR-20a* + *miR-191*	*miR-20a* + *miR-26a*	*miR-20a* + *miR-26a*	*miR-20a* + *miR-26a* + *miR-191*
WT	*miR-20a* + *miR-26a*	*miR-20a* + *miR-25*	*miR-25* + *miR-26a*	*miR-25* + *miR-26a*	*miR-20a* + *miR-25*	*miR-20a* + *miR-25*	*miR-20a* + *miR-25*	*miR-20a* + *miR-25* + *miR-26a*
SC	*miR-25* + *miR-191*	*miR-25* + *miR-191*	*miR-25* + *miR-191*	*miR-25* + *miR-191*	*miR-25* + *miR-191*	*miR-25* + *miR-191*	*miR-25* + *miR-191*	—
CO	*miR-20a* + *U6*	*miR-26a* + *miR-191*	*miR-20a* + *miR-26a*	*miR-20a* + *miR-26a*	*U6* + *miR-25*	*miR-20a* + *miR-26a*	*miR-20a* + *miR-26a*	*miR-20a* + *miR-25* + *miR-26a or miR-20a* + *miR-26a* + *miR-191 or miR-20a* + *miR-26a* + *U6*

All, all samples; CY, cystic; NC, non-cystic; HT, haploinsuficient; WT, wild-type; SC, severely cystic phenotype; CO, severely cystic phenotype controls.

GeNorm indicated that *miR-20a*, *miR-25*, *miR-26a, miR-191* and *U6* presented M values below 1.5 between the tissue groups, except for HT tissues that showed high variability (M > 1.5) for *U6* (Supplementary Table 2).

The evaluation of relative expression stability by the BestKeeper software defines the genes that display SD higher than 1.0 as unstable. Again, *miR-20a*, *miR-25* and *miR-26a* presented SD lower than 1.0 and CV below 3.0 in the All, CY, NC, HT and WT groups (Supplementary Table 2). However, *U6* showed SD below 1.0 only for the SC and CO groups, thus it is not a suitable housekeeping gene for the All, CY, NC, HT and WT sample groups (Supplementary Table 2). Based on these results, *miR-20a*, *miR-25* and *miR-26a* were ranked as the most stable candidate housekeeping genes, whereas *miR-191* and *U6* were deemed least stable.

### Analysis of the best combination of housekeeping miRNAs

The GeNorm software package recommends at least two genes as combination of housekeeping genes. Table 2 shows the best combination of housekeeping miRNAs for each model/control group pair, based on the different software packages and a visual inspection of all ranks generated by such analyses. The comparisons of *Pkd1*-deficiency kidney tissues versus their respective controls identified *miR-20a* for the CY and NC group pair, *miR-20a* and *miR-26a* for HT and WT, and *miR-25* and *miR-26a* for SC and CO as the most stable housekeeping genes. The comparison including all groups, in turn, revealed the *miR-20a* + *miR-26a* pair as the most stable housekeeping gene selection (Table 2).

**Figure 3:**
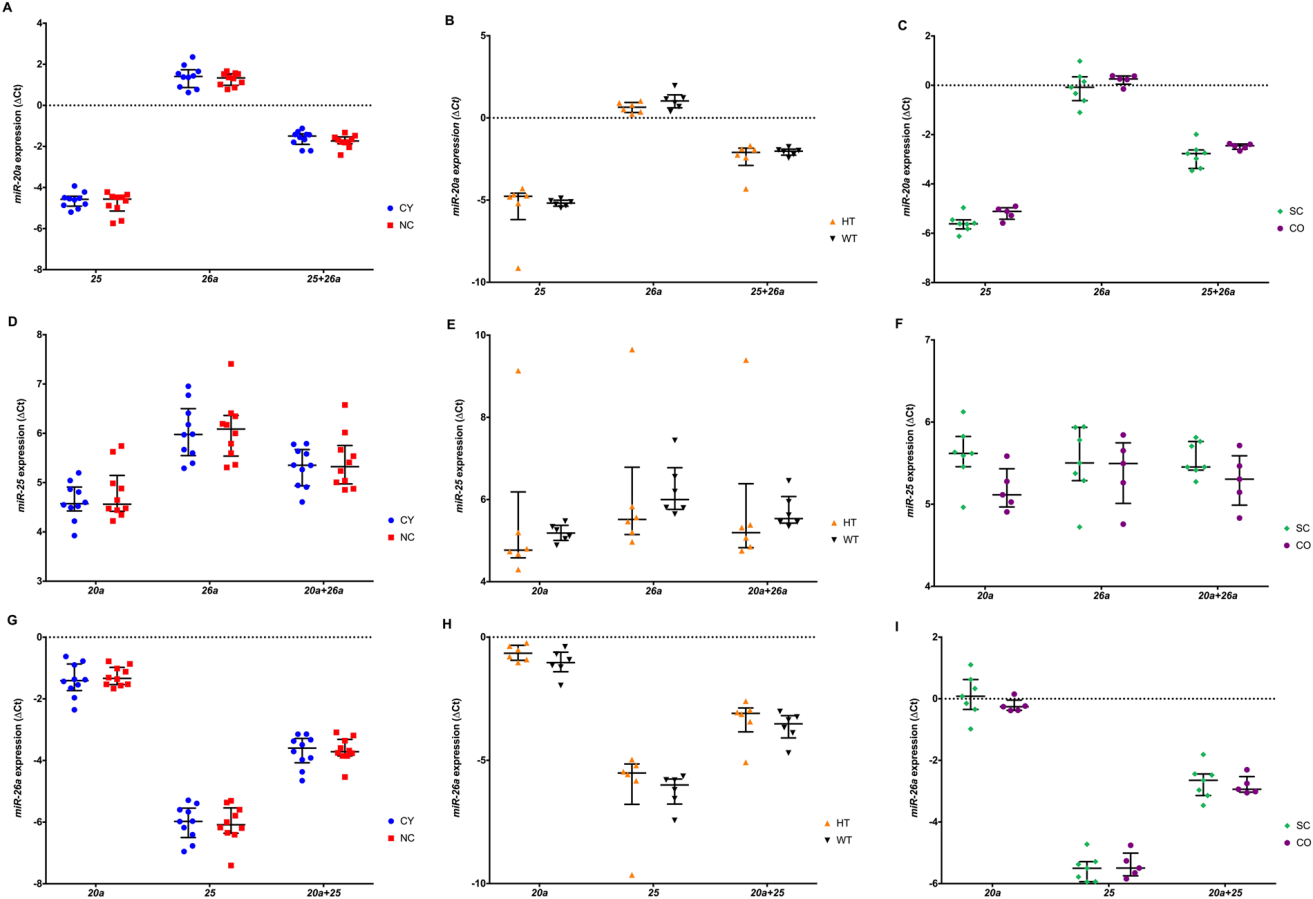
The ΔCt values of *miR-20a* (**A**–**C**), *miR-25* (**D**–**F**) and *miR-26a* (**G**–**I**) candidate housekeeping genes normalized by different combinations of each other. A lower threshold value (Ct) indicates a higher gene expression. The median values are expressed as horizontal lines, and the error bars represent interquartile range. CY, cystic; NC, non-cystic; HT, haploinsufficient; WT, wild-type; SC, severely cystic phenotype; CO, severely cystic phenotype controls. *20a*, target expression normalized by *miR-20a*; *25*, target expression normalized by *miR-25*; *26a*, target expression normalized by *miR-26a*; *25* + *26a*, target expression normalized by *miR-25* + *miR-26a*; *20a* + *25*, target expression normalized by *miR-20a* + *miR-25*; *20a* + *26a*, target expression normalized by *miR-20a* + *miR-26a*. *p < 0.008 by Mann-Whitney test, followed by Bonferroni correction.

To evaluate the effects of the best candidate housekeeping genes determined by the different algorithms, the expression levels of the top three candidate miRNAs (*miR-20a*, *miR-25* and *miR-26a*) were normalized by each other (Fig. 3). *miR-20a*, *miR-25* and *miR-26a* did not differ between the groups ranking for the best ones when normalized by each other (Fig. 3): All comparisons showed no statistically differential expression, but when *miR-20* and *miR-26* were used as housekeeping genes we observed a more cohesive distribution and equivalent expression in the CY vs NC, HT vs WT and SC vs CO group pairs These results indicate that *miR-20* and *miR-26a* are the most suitable genes to be used together as housekeeping genes in the assessed experimental scenario.

### Determination of the suitable number of housekeeping genes

**Figure 4:**
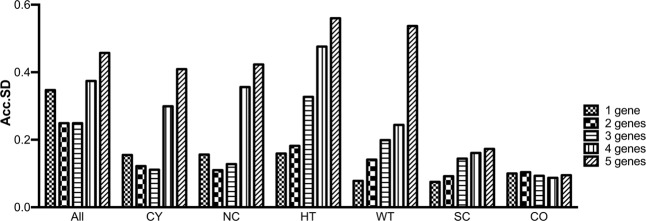
Optimal number of reference genes according to GenEx analyses. Accumulated standard deviation (Acc.SD) for the five candidate housekeeping genes in all groups of samples allows to estimate the ideal number of genes for normalization.Acc.SD was calculated with the GenEx software package. Lower values of Acc.SD indicate the optimal number of reference genes. CY, cystic; NC, non-cystic; HT, *Pkd1*-haploinsufficient; WT, wild-type; SC, severely cystic phenotype; CO, severely cystic phenotype controls.

After ranking the candidate housekeeping genes according to their stability, a second approach was applied to determine the optimal number of reference genes to be used in each dataset. This analysis was performed using the GenEx software package. The optimal number of reference genes was calculated using the Acc.SD for the five candidate housekeeping genes (Fig. 4). Estimating the use of the ideal number of genes for normalization, one gene seems to be the best number in HT (*mir-26a*), WT (*mir-20a*), SC (*mir-25*), and CO (*mir-26a*) groups. The analysis showed that two (*mir-20a* + *mir-26a*) is the optimal number of references to be considered for normalization of miRNAs gene expression when using all the samples and the NC group. Three (*mir-20a* + *mir-25* + *mir-26a*), in turn, was the optimal number of housekeeping genes to be applied to the CY group. We did not observe difference among the numbers of housekeeping genes to be used for the CO group.

### Correlation between the top three candidate housekeeping miRNAs expression

**Figure 5:**
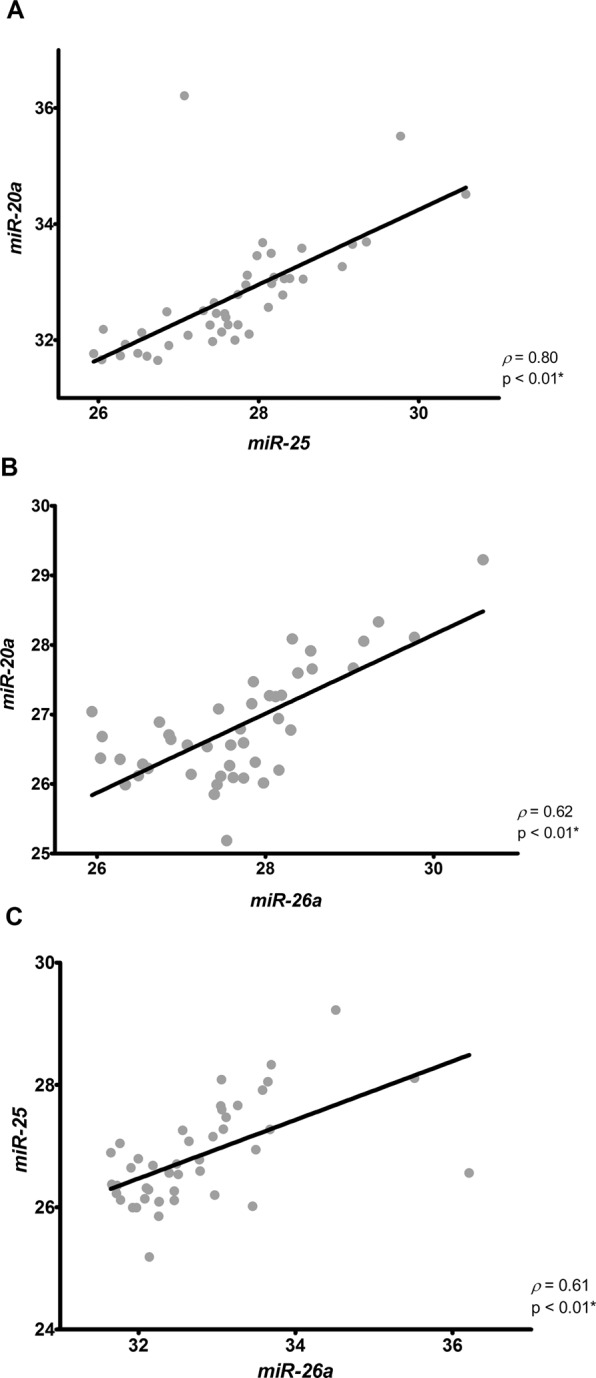
Correlation matrix between the expression of *miR-20a* and *miR-25* (**A**), *miR-20a* and *miR-26a* (**B**), and between *miR-25* and *miR-26a* (**C**) candidate housekeeping genes. *ρ*: Spearman’s rank correlation coefficient. *p < 0.05.

Correlation analyses were performed using the miRNAs expression data from all evaluated kidney samples. The expression level of the three best candidate housekeeping genes demonstrated a strong correlation between *miR-20a* and *miR-25* (*ρ* = 0.80, p < 0.001, Fig. 5). Additionally, a moderate correlation was observed between *miR-20a* and *miR-26a* (*ρ* = 0.62, p < 0,001, Fig. 5), as well as between *miR-25* and *miR-26a* (*ρ* = 0.61, p < 0,001, Fig. 5). These results suggest that, in addition to *miR-25* and *miR-26a* having shown a moderate correlation, they are still correlated in all the samples herein evaluated and can be used together as suitable housekeeping genes. Our results showed that CT data dispersion of *miR-20a* and *miR-26a* increases substantially after normalization by *U6*. These results, were represented in Supplementary Fig. 3.

### Validation of the best candidate housekeeping genes for normalizing *miR-17*, *miR-21* and *let-7a* target genes

**Figure 6:**
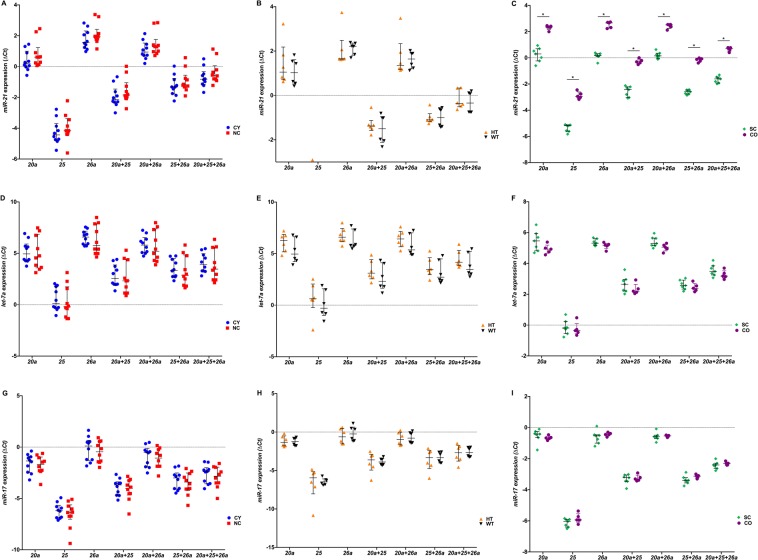
The ΔCt values of *miR-21* (**A**–**C**), *let-7a* (**D**–**F**) and *miR-17* (**G**–**I**) target genes normalized by different combinations of the three best candidate housekeeping genes (*miR-20a*, *miR-25 and miR-26a*). A lower threshold value (Ct) indicates a higher gene expression. The median values are represented as horizontal lines, and the error bars represent interquartile range. CY, cystic; NC, non-cystic; HT, haploinsufficient; WT, wild-type; SC, severely cystic phenotype; CO, severely cystic phenotype controls. *20a*, target expression normalized by *miR-20a*; *25*, target expression normalized by *miR-25*; *26a*, target expression normalized by *miR-26a*; *25* + *26a*, target expression normalized by *miR-25* + *miR-26a*; *20a* + *25*, target expression normalized by *miR-20a* + *miR-25*; *20a* + *26a*, target expression normalized by *miR-20a* + *miR-26a*, target expression normalized by *miR-20a* + *miR-25* + *miR-26a*. *p < 0.008 by Mann-Whitney test, followed by Bonferroni correction.

In order to validate the three best candidate housekeeping genes stability, the relative expression of *miR-17*, *miR-21* and *let-7a* target genes was assessed using different combinations of *miR-20a*, *miR-25* and *miR-26a* (Fig. 6). The expression levels of *miR-21* were consistent with upregulation in SC compared to its control CO when normalized by different combinations of the best candidate housekeeping genes (Fig. 6C), trends of upregulation was observed in the CY and HT groups when compared with their respective controls (Fig. 6A,B). Trends of *let-7a* downregulation in CY, HT and SC compared to its control kidneys, was observed when their expression was normalized by different combinations of *miR-20a*, *miR-25* and *miR-26a* (Fig. 6D,F). Moreover, *miR-17* did not present expression statistically different in the CY vs NC, HT vs WT and SC vs CO groups (Fig. 6G,I).

When *U6* was used as housekeeping gene, the target genes did not differ between CY vs NC and HT vs WT, except for *miR-21* that showed downregulation in CY vs CO, HT vs CO and SC vs CO (Supplementary Fig. 2). These results also suggest that the SC group and its respective control CO have an expression profile slightly different from the other studied ADPKD-related mouse models. Taken together, the use of *miR-25* and *miR-26a* showed to be the most suitable pair of housekeeping genes among all considered sample groups.

To evaluate whether the severity of renal cystic phenotype was a significant modifier of expression of potential housekeeping genes, we investigated potential correlations between the expression levels of *miR-20a*, *miR-25* and *miR-26a* and kidney weight/body weight (KW/BW) in the CY group. The expression levels of *miR-20a*, *miR-25* and *miR-26a* were not correlated with the KW/BW (*ρ* = −0.52, p < 0,18, Supplementary Fig. 3A; *ρ* = −0.24, p < 0,57, Supplementary Fig. 3B; and *ρ* = −0.14, p = 0.74, Supplementary Fig. 3C, respectively). The lack of correlation observed between the expression of the evaluated candidate housekeeping genes and kidney weight suggests that the cystic burden does not significantly influence the expression level of the analyzed housekeeping genes. These results provide support to use these miRNAs as controls in studies involving animals with different severities of renal cystic phenotype.

## Discussion

In recent years, miRNAs have emerged as key players in tightly-controlled biological processes such as proliferation^37,41,42^, apoptosis^43–46^ and metabolism^47^. MiRNAs are known to be deregulated in numerous kidney diseases including ADPKD^41–43^ and there is evidence to suggest that miRNA expression profiles may be more accurate in classifying kidney disease progression than mRNA expression profiles^11^. The relative miRNA expression quantification is usually compared with a stably expressed housekeeping miRNA from the same sample^33,48^. The high sensitivity of RT-qPCR demands appropriate normalization to correct for non-biological variation and the use of housekeeping genes remains the most commonly used method^49^. However, there are no universally accepted housekeeping transcripts for miRNA data normalization by RT-qPCR analyses. In this scenario, the present study describes the first assessment of candidate housekeeping genes for the normalization of miRNA expression by RT-qPCR in kidney tissue of mouse models with distinct patterns of *Pkd1* deficiency. This is a significant problem to be solved, since different animal models orthologous to ADPKD have been and will keep being used to address distinct questions related to ADPKD pathogenesis and interventional studies.

Four candidate miRNA housekeeping genes (*miR-20a*, *miR-25*, *miR-26a* and *miR-191*) and the *U6* small nuclear RNA were selected from previous studies^33,37–39,50–52^. We used six algorithms (NormFinder, GeNorm, BestKeeper, DataAssist, the comparative ΔCt method and RefFinder) to identify the suitable housekeeping genes for relative quantitation of miRNA in fresh-frozen kidney tissues. Each algorithm ranked the best candidate reference genes. The software packages recommended *miR-20a*, *miR-25* and *miR-26a* as the most stable housekeeping genes among the tissue groups.

The expression of small nuclear (snRNAs) and nucleolar (snoRNAs) may exhibit tissue-specific and developmental regulation^53^, emphasizing the need for validation of commercially-available control assays. Although *U6* is the most commonly used gene to normalize miRNA RT-qPCR data^37–39^, we showed that it was the least stable candidate housekeeping gene in the evaluated samples. This result was also observed in other tissues and diseases^35,36,54,55^.

Since *miR-20a*, *miR-25* and *miR-26a* were the most suitable candidate genes, they were selected for normalization of *miR-17*, *miR-21* and *let-7a* target genes. The differences in *miR-21* expression detected between the tissue groups markedly varied depending on which single housekeeping gene was used for normalization. These results corroborate previous studies that associated *miR-21* with increase in cystogenesis and kidney size^20,38^. On the other hand, Gee *et al*. observed that *miR-21* presented variable expression in breast cancer and head and neck squamous cell carcinoma samples when normalized by *U6*, *U44*, *U48* and *U43*^54^. In renal cell carcinoma, *miR-28*, *miR-103* and *miR-106a* were proven to be more stably expressed than *U6*^56^. Therefore, these findings indicate that the use of unvalidated housekeeping genes may lead to inaccurate and unreliable results, and the use of snRNAs for normalization of expression of miRNAs might introduce bias in the associations between miRNA and the pathology or outcome^50,54^.

The current observed differences in *miR-17* expression detected between the tissue groups were at variance with many studies^21,24,37,47^, depending on which single housekeeping gene and animal models with *Pkd1* deficiency had been used for normalization. These findings draw attention to the potential effects of the housekeeping gene choice on the outcome of a study and demonstrates the need for validation of candidate housekeeping genes to generate reliable expression data. Even though our analysis suggested that the SC group and its respective control CO have an expression profile slightly different from the other *Pkd1*-deficient models, the use of *miR-25* and *miR-26a* showed to be the most suitable pair of housekeeping genes among all.

Finally, we assessed the correlation between the best candidate housekeeping genes and KW/BW ratio in the CY group of samples. The detected absence of correlation between the expression levels of the best ones and the KW/BW ratio in the CY group suggests that the level of cystic involvement does not lead to significant changes in housekeeping gene expression. Therefore, *miR-20a*, *miR-25* and *miR-26a* can be used as housekeeping genes in groups that include different stages of cystic burden in murine models orthologous to ADPKD.

One essential observation is the careful selection and validation of appropriate reference genes as a basis for normalizing the variability between samples in the corresponding study designs to each animal model used in the literature for experimental ADPKD research, amongst them *Ksp/cre* mice, *Pkhd1/cre* mice, *Kif3a* ^flox/flox^ mice, *Pkd2* ^flox/flox^ mice, *Pkhd1*^−/−^ mice, *Hnf-1β* ^flox/flox^ mice, *miR-17∼92*^flox/flox^.

Based on current findings, we propose an appropriate selection of the best housekeeping genes for each comparison involving one or more mouse models, following the approach adopted in the present study. This procedure consisted in evaluating reference genes in three specific mouse models, namely *Pkd1*^flox/flox^:*Nestin*^cre^(CY) *Pkd1*^+/−^ (HT) and *Pkd1*^V/V^ (SC). The best housekeeping gene selection for each model/control group pair should be used when comparing a specific model with its respective control, while in comparisons including more than one model the best choice should be based on the whole analysis. Such specific approach enables a more proper and reliable normalization for future studies of dysregulated miRNAs within the ADPKD progression cascade. This task obviously implies reliable comparisons between expression data in different stages of the disease, including mild to severe PKD.

One limitation of the present study relies on the selection of only 5 candidate housekeeping genes for validation based on previous studies, but we recognize that more and other genes could also be suitable for accurate miRNA expression normalization by RT-qPCR in ADPKD in in kidney tissue samples of orthologous mouse models and that the analysis of candidate housekeeping genes should be further tested using other models as well.

Previous studies suggested that, unlike mRNAs, the miRNA fraction present in FFPE tissues is relatively unaffected by the fixation process and that miRNAs extracted from these tissues may be accurately profiled using RT-qPCR^57–59^. In this context, the housekeeping genes identified in this study may also prove useful for miRNA RT-qPCR analysis of FFPE kidney tissues. Our findings will allow further analysis in kidney tissue of *Pkd1*-deficient mouse models gene expression to elucidate the role of different regulatory miRNAs in different scenarios of or related to ADPKD.

Normalizing to a suitable housekeeping gene, therefore, can eliminate differences due to sampling and quality of RNA and identify real changes in miRNA expression levels. In the present study, we analyzed a series of candidates and identified the most suitable housekeeping genes to be used for miRNA expression normalization in RT-qPCR studies in kidney tissues of mouse models orthologous to ADPKD and their respective controls.

The housekeeping gene spectrum and data generated by our work should therefore be employed in miRNA-related studies involving *Pkd1*-deficient mouse models. Among the genes currently used in this field, appropriate combinations of the *miR-20a*, *miR-25*, and *miR-26a* housekeeping genes offer increased accuracy and resolution in the quantitation of gene expression data, favoring the detection of smaller changes in miRNA expression than otherwise possible. The proper selection of the best housekeeping genes to be used in each of these scenarios should follow the guidelines specific to each comparison. It must be noted that other *Pkd1*-deficient models distinct from the ones analyzed in the current study may display slight expression differences of reference genes. Even in these cases, however, our guidelines would still be the best available housekeeping controls for such studies.

## Methods

### ADPKD mouse models

We used three mouse models with distinct *Pkd1*-deficiency profiles in the current study, all generated and maintained on the C57BL/6 background. Only kidneys from male animals were analyzed in order to avoid potential gender-related experimental variability. Two models were evaluated at 10-12 weeks of age, including a renal cystic mouse (*Pkd1*^flox/flox^:*Nestin*^cre^, CY, n = 10) and its corresponding non-cystic control (*Pkd1*^flox/flox^, NC, n = 10), and a *Pkd1*-haploinsufficient mouse (*Pkd1*^+/−^, HT, n = 6) and its respective wild-type control (*Pkd1*^+/+^, WT, n = 6). The third model was assessed at 15 days of life due to its severely renal cystic phenotype (*Pkd1*^V/V^, SC, n = 7) along with its wild-type control (CO, n = 5). These animals were genotyped using specific PCRs. The CY mouse is homozygous for a *Pkd1*-floxed allele (*Pkd1*^flox^) and displays a mosaic pattern of gene inactivation, induced by a Nestin-Cre transgene through excision of exons 2–4 (*Pkd1*^flox/flox^:Nestin^cre^)^25,27–29^. The HT model is heterozygous for a *Pkd1* null allele, characterized by early transcriptional interruption^25,26^, and develops no renal cysts by 12 weeks of age. The SC model is homozygous for the *Pkd1* knockin T3041V allele, which prevents the autoproteolytic cleavage of PC1 at the GPS site^25,30^. *Pkd1*^V/V^ animals have no gross phenotype by postnatal day (P) 6 but develop rapid and progressive distal nephron dilatation thereafter. This severe renal phenotype, that eventually leads to renal failure, is apparently responsible for the early mortality that occurs between the 2nd and 6th week^30^. Animals were fed *ad libitum* and housed at constant ambient temperature in a 12-hour light cycle.

Animal procedures were approved by the Internal Biosafety Commission of Genetically Modified Organisms of the University of São Paulo School of Medicine and by the Universidade Federal de São Paulo (UNIFESP) Research Ethics Committees.

SC (*Pkd1*^V/V^) and its wild-type control (CO) animals were euthanized by adopting cervical dislocation and the other animal groups were euthanized with intraperitoneal thiopental (0.4 mg/g of body weight); their kidneys were appropriately harvested for RT-qPCR analyses. All experiments were conducted in accordance with international standards of animal care and experimentation. Both kidneys were collected and stored at −80 °C for further use.

### Housekeeping genes

We selected these genes based on the observation that they were among the miRNAs with the lowest variances in a previous study of ours (TLDA Taqman Array, unpublished data) and that miRNA expression studies showed minor evidence of differential expression^10,20,37–39^. These genes not only behaved stably but also presented expression stability in transcriptomic analysis. Of note, *U6* and *miRNA191* presented stable expression, also documented in a previous report^33^. All these five miRNAs are constitutively expressed in kidney tissue of mouse models orthologous to ADPKD, have independent cellular functions, and are assumed not to be co-regulated.

### RNA extraction

Renal tissue lysis was performed using zirconia beads (Interprise, USA) and the Precellys (BioAmerica, USA) homogenizer. TRIzol (Life Technologies, USA) was employed for total RNA extraction according to the manufacturer’s protocol. The RNA quantity and purity were determined using the NanoVue spectrophotometer (GE Healthcare Life Sciences, USA) and analyzed with the Agilent 2100 Bioanalyzer 6000 Nanochip (Agilent Technologies Inc., Waldbronn, BW, Germany). Total RNA was stored at −80 °C until further use.

### cDNA preparation and RT-qPCR

Complementary DNA (cDNA) was synthesized from 1 µg of total RNA with an oligonucleotide pool for each evaluated gene (Supplementary Table 1), using the TaqMan® microRNA reverse transcription kit (Life Technologies) according to the manufacturer’s instructions. Detection of the expression range of the evaluated genes was achieved by using TaqMan® assays and the QuantStudio® 7 Flex real-time PCR system (Applied Biosystems, USA). The used primers are shown in Supplementary Table 1. *miR-17*, *miR-21* and *let-7a* were employed as target genes^20–24^. All reactions were run in triplicate. The expression of the candidate housekeeping genes is represented by the original cycle threshold (Ct) value.

### Analysis of housekeeping gene expression stability

Cts of RT-qPCR were manually settled as 0.02 while the mean Ct values of the three technical replicates were imported to six algorithms (Fig. 1): NormFinder (version 0.953; https://moma.dk/normfinder-software)^60^, GeNorm (https://genorm.cmgg.be/)^40^, BestKeeper (version 1.0; https://www.gene-quantification.de/bestkeeper.html)^61^, DataAssist^TM^ (version 3.01; https://www.thermofisher.com/br/en/home/technical-resources/software-downloads/dataassist-software.html), the comparative ΔCt method^62^ and RefFinder (https://www.heartcure.com.au/reffinder/)^63^, following the authors’ recommendations. These software packages were used to determine the relative expression stability of the candidate housekeeping genes and to generate a ranking for the best ones.

The optimal number of reference genes was selected using the GenEx software package. GenEx is a software for the processing and analysis of RT-qPCR data and provides methods to select and validate housekeeping genes, classify samples, group genes and monitor time-dependent processes. GenEx calculates Accumulated standard deviation (Acc.SD); lower Acc.SD values, in turn, indicate the optimal number of reference genes (https://www.biomcc.com/genex-software.html).

### Statistical analysis

The Shapiro-Wilk test showed that the Ct values of the candidate housekeeping genes were not normally distributed. Therefore, results are shown in median and interquartile range (IQ). Initially, the Kruskal-Wallis test was used to evaluate possible expression differences of *miR-17*, *miR-20a*, *miR-21*, *miR-25*, *miR-26a*, *miR-191*, *U6*, and *let-7a* among the sample groups. When the Kruskal-Wallis test showed significance (p < 0,05), the Mann-Whitney test, followed by Bonferroni correction, was used for all comparisons between two groups. Lastly, the Spearman correlation test was used to verify potential correlations among the expression levels of *miR-20a*, *miR-25* and *miR-26a*, and between the expression levels of these miRNAs in all kidney samples evaluated herein and kidney weight. A value between 0.30–0.50 was determined as a weak correlation, 0.50-0.70 as moderate, 0.70-0.90 as strong, and 0.90–1.00 as a very strong correlation^64^.

## Supplementary information

<b>Supplementary Information: Identification of housekeeping genes for microRNA expression analysis in kidney tissues of <i>Pkd1</i> deficient mouse models</b>.

## Data Availability

All data including supporting datasets are made available as main figures or Supplementary Information Files.
