# Supplementary material for: Identification of housekeeping genes for microRNA expression analysis in kidney tissues of Pkd1 deficient mouse models
Source: Sci Rep. 2020 Jan 14;10:231. doi: 10.1038/s41598-019-57112-4 (PMC6959247; doi:10.1038/s41598-019-57112-4)
Supplement: Supplementary file 1 — Supplementary Information: Identification of housekeeping genes for microRNA expression analysis in kidney tissues of Pkd1 deficient mouse models. [file 41598_2019_57112_MOESM1_ESM.doc]

Manuscript Title:

**Identification of housekeeping genes for microRNA expression analysis in kidney tissues of *Pkd1* deficient mouse models**

Muñoz JJ^1^*, Anauate AC^1^*, Amaral AG^2^, Ferreira FM^3^, Meca R^1^, Ormanji MS^1^, Boim MA^1^, Onuchic LF^2^ & Heilberg IP^1^

^1^Nephrology Division, Department of Medicine, Universidade Federal de São Paulo, São Paulo, Brazil.

^2^Divisions of Molecular Medicine and Nephrology, University of São Paulo School of Medicine, São Paulo, Brazil.

^3^Laboratory of Immunology, Heart Institute, University of São Paulo School of Medicine.

*These authors contributed equally.

**Corresponding author:**

Ita Pfeferman Heilberg, M.D., PhD

Nephrology Division

Universidade Federal de São Paulo

Rua Botucatu 740 - Vila Clementino

04023-900

São Paulo - Brazil

Tel + 5511-55764848 ext 2465

Fax + 5511-59041684

E-mail address: [ita.heilberg@gmail.com](mailto:ita.heilberg@gmail.com)

**Running title:** MicroRNA housekeeping genes in *Pkd1* deficient mouse models.

**Table of Contents:**

**Supplementary Information**

1. **Supplementary Figure 1**
2. **Supplementary Figure 2**
3. **Supplementary Figure 3**
4. **Supplementary Table 1**
5. **Supplementary Table 2**





**Supplementary Figure 1.** The ΔCt values of *miR-20a* (**A**), *miR-25* (**B**) and *miR-26a* (**C**) candidate housekeeping genes normalized by *U6*. A lower threshold value (Ct) indicates a higher gene expression. The median values are expressed as horizontal lines, and the error bars represent interquartile range. CY, cystic; NC, non-cystic; HT, haploinsufficient; WT, wild-type; SC, severely cystic phenotype; CO, severely cystic phenotype controls. *U6*, target expression normalized by *U6*. *p<0.008 by Mann-Whitney test, followed by Bonferroni correction.





**Supplementary Figure 2.** The ΔCt values of *miR-21* (**A**), *let-7a* (**B**) and *miR-17* (**C**) target genes normalized by *U6*. A lower threshold value (Ct) indicates a higher gene expression. The median values are represented as horizontal lines, and the error bars represent interquartile range. CY, cystic; NC, non-cystic; HT, haploinsuficient; WT, wild-type; SC, severely cystic phenotype; CO, severely cystic phenotype controls. *p<0.008 by Mann-Whitney test, followed by Bonferroni correction.

**
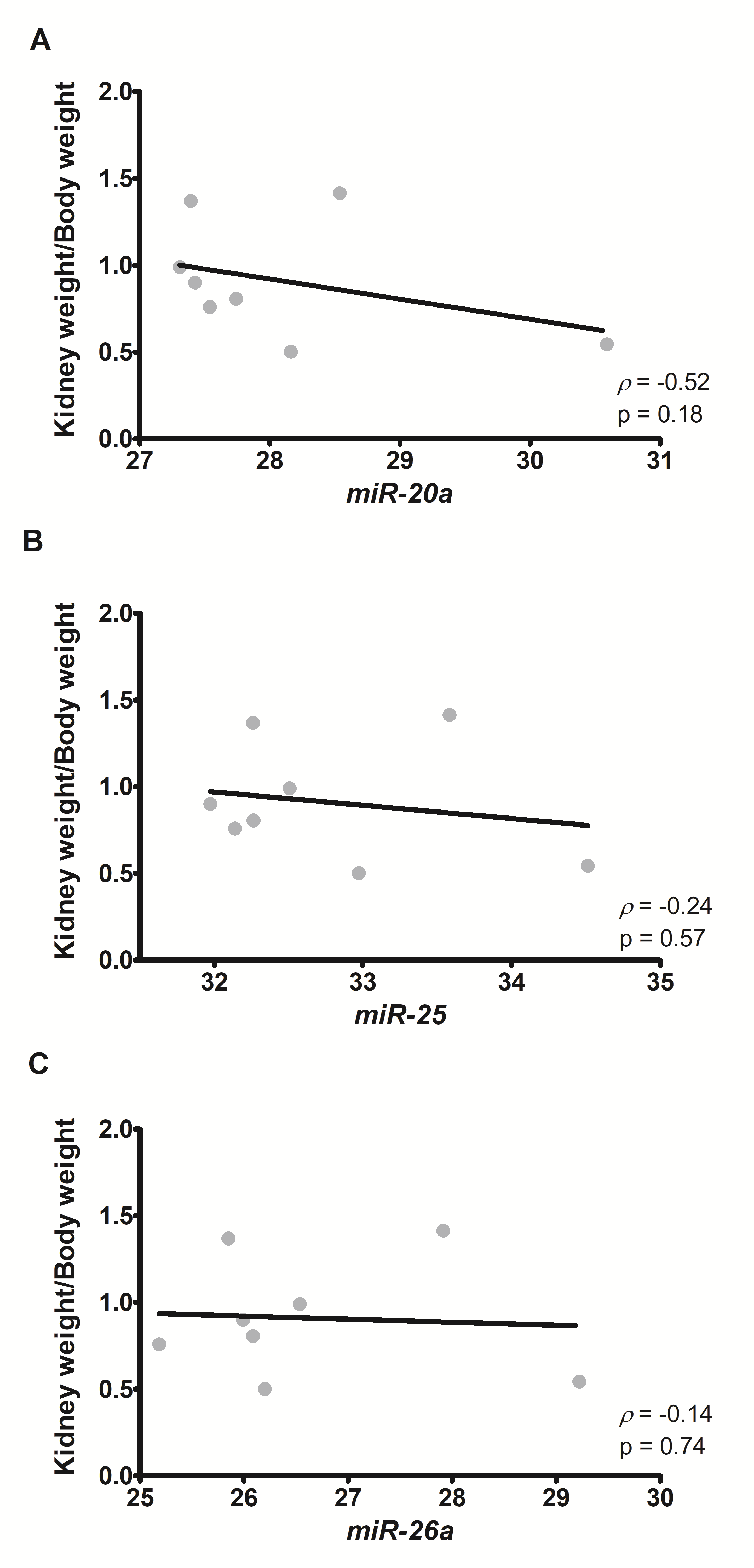
**

**Supplementary Figure 3**. Correlation matrix between the expression of *miR-20a* (**A**), *miR-25* (**B**), and *miR-26a* (**C**) candidate housekeeping genes and the kidney weight/body weight ratio. *ρ*: Spearman’s rank correlation coefficient. *p<0.05.

**Supplementary Table 1**. List of primers of the housekeeping genes.

| **Gene symbol** | **Gene name** | **Assay ID*** | **Gene function** |
| --- | --- | --- | --- |
| *miR-17* | *mmu-miR-17-5p* | 2308 | Target gene |
| *miR-20a* | *miR-20a-5p* | 0508 | Housekeeping |
| *miR-21* | *mmu-miR-21a-5p* | 0397 | Target gene |
| *miR-25* | *miR-25-3p* | 0403 | Housekeeping |
| *miR-26a* | *miR-26a-5p* | 0405 | Housekeeping |
| *miR-191* | *miR-191* | 0490 | Housekeeping |
| *let-7a* | *mmu-let-7a-5p* | 0377 | Target gene |
| *U6* | U6 small nuclear RNA | 1973 | Housekeeping |

*TaqMan probes were purchased as Assays-on-Demand Products (Applied Biosystems).

**Supplementary Table 2.** Ranking of the five candidate housekeeping miRNAs determined by each of the six algorithms selected.

|  | **NormFinder** | **SD** | **GeNorm** | **M value** | **RefFinder** | **Geomean** | **ΔCt method** | **Mean SD** | **Bestkeeper** | **CV** | **SD** | **DataAssist** | **Score** |
| --- | --- | --- | --- | --- | --- | --- | --- | --- | --- | --- | --- | --- | --- |
| **All** | *miR-26a** | 0,32 | *miR-26a* | 0,71 | *miR-26a* | 1,19 | *miR-26a* | 1,12 | *miR-191* | 1,70 | 0,43 | *miR-26a* | 1,06 |
|  | *miR-25* | 0,37 | *miR-20a* | 0,71 | *miR-20a* | 2,00 | *miR-20a* | 1,15 | *miR-26a* | 2,39 | 0,64 | *miR-20a* | 1,11 |
|  | *miR-20a** | 0,43 | *miR-25* | 0,78 | *miR-191* | 2,83 | *miR-25* | 1,23 | *miR-25* | 2,18 | 0,71 | *miR-25* | 1,26 |
|  | *miR-191* | 0,95 | *miR-191* | 0,98 | *miR-25* | 3,00 | *miR-191* | 1,57 | *miR-20a* | 2,72 | 0,75 | *miR-191* | 1,63 |
|  | *U6* | 0,98 | *U6* | 1,29 | *U6* | 5,00 | *U6* | 1,82 | *U6* | 7,64 | 1,94 | *U6* | 1,77 |
| **CY** | *miR-26a* | 0,15 | *miR-25* | 0,38 | *miR-20a* | 1,32 | *miR-20a* | 0,82 | *miR-191* | 1,53 | 0,39 | *miR-20a* | 0,82 |
|  | *miR-20a* | 0,19 | *miR-20a* | 0,38 | *miR-25* | 1,86 | *miR-25* | 0,90 | *miR-25* | 1,90 | 0,62 | *miR-25* | 0,87 |
|  | *miR-25* | 0,23 | *miR-26a* | 0,49 | *miR-191* | 2,83 | *miR-26a* | 0,90 | *miR-20a* | 2,70 | 0,76 | *miR-26a* | 0,93 |
|  | *miR-191* | 1,15 | *miR-191* | 0,71 | *miR-26a* | 2,91 | *miR-191* | 1,15 | *miR-26a* | 3,83 | 1,03 | *miR-191* | 1,29 |
|  | *U6* | 1,66 | *U6* | 1,08 | *U6* | 5,00 | *U6* | 1,65 | *U6* | 7,98 | 2,06 | *U6* | 1,72 |
| **NC** | *miR-26a* | 0,16 | *miR-26a* | 0,31 | *miR-20a* | 1,57 | *miR-20a* | 0,86 | *miR-191* | 1,40 | 0,35 | *miR-20a* | 0,83 |
|  | *miR-20a* | 0,16 | *miR-20a* | 0,31 | *miR-26a* | 1,68 | *miR-26a* | 0,88 | *miR-20a* | 2,33 | 0,66 | *miR-26a* | 0,84 |
|  | *miR-25* | 0,32 | *miR-25* | 0,48 | *miR-25* | 2,71 | *miR-25* | 1,05 | *miR-25* | 2,46 | 0,81 | *miR-25* | 0,96 |
|  | *miR-191* | 1,37 | *miR-191* | 0,80 | *miR-191* | 3,34 | *U6* | 1,56 | *miR-26a* | 2,76 | 0,75 | *miR-191* | 1,46 |
|  | *U6* | 1,57 | *U6* | 1,10 | *U6* | 4,23 | *miR-191* | 1,63 | *U6* | 7,46 | 1,90 | *U6* | 1,63 |
| **HT** | *miR-26a* | 0,16 | *miR-26a* | 0,32 | *miR-20a* | 1,00 | *miR-20a* | 1,24 | *miR-191* | 1,31 | 0,33 | *miR-26a* | 1,17 |
|  | *miR-20a* | 0,33 | *miR-20a* | 0,32 | *miR-26a* | 1,86 | *miR-26a* | 1,35 | *miR-20a* | 1,44 | 0,40 | *miR-20a* | 1,18 |
|  | *miR-191* | 0,91 | *miR-191* | 0,51 | *miR-191* | 2,71 | *miR-191* | 1,64 | *miR-26a* | 1,79 | 0,49 | *miR-191* | 1,38 |
|  | *miR-25* | 1,63 | *miR-25* | 1,10 | *miR-25* | 4,00 | *miR-25* | 2,05 | *miR-25* | 2,99 | 0,99 | *miR-25* | 1,95 |
|  | *U6* | 2,05 | *U6* | 1,53 | *U6* | 5,00 | *U6* | 2,18 | *U6* | 6,94 | 1,86 | *U6* | 2,24 |
| **WT** | *miR-20a* | 0,08 | *miR-25* | 0,21 | *miR-25* | 1,86 | *miR-26a* | 1,13 | *miR-20a* | 0,53 | 0,15 | *miR-20a* | 0,90 |
|  | *miR-26a* | 0,27 | *miR-20a* | 0,21 | *miR-26a* | 2,00 | *miR-25* | 1,15 | *miR-25* | 0,66 | 0,22 | *miR-25* | 0,96 |
|  | *miR-25* | 0,53 | *miR-191* | 0,29 | *miR-20a* | 2,06 | *miR-20a* | 1,19 | *miR-191* | 1,02 | 0,26 | *miR-26a* | 1,01 |
|  | *miR-191* | 0,77 | *miR-26a* | 0,46 | *miR-191* | 2,63 | *miR-191* | 1,35 | *miR-26a* | 1,50 | 0,40 | *miR-191* | 1,05 |
|  | *U6* | 2,50 | *U6* | 1,18 | *U6* | 5,00 | *U6* | 2,59 | *U6* | 8,71 | 2,28 | *U6* | 2,52 |
| **SC** | *miR-25* | 0,07 | *miR-25* | 0,15 | *miR-191* | 1,00 | *miR-191* | 0,64 | *miR-25* | 0,74 | 0,24 | *miR-25* | 0,38 |
|  | *miR-191* | 0,17 | *miR-191* | 0,15 | *miR-25* | 2,11 | *miR-25* | 0,65 | *miR-191* | 0,93 | 0,24 | *miR-191* | 0,41 |
|  | *miR-20a* | 0,39 | *miR-20a* | 0,32 | *miR-20a* | 3,22 | *miR-20a* | 0,70 | *miR-26a* | 1,10 | 0,29 | *miR-20a* | 0,53 |
|  | *miR-26a* | 0,48 | *miR-26a* | 0,40 | *U6* | 3,36 | *U6* | 0,76 | *miR-20a* | 1,33 | 0,35 | *miR-26a* | 0,57 |
|  | *U6* | 0,58 | *U6* | 0,50 | *miR-26a* | 4,40 | *miR-26a* | 0,87 | *U6* | 1,46 | 0,34 | *U6* | 0,65 |
| **CO** | *miR-20a* | 0,10 | *miR-26a* | 0,16 | *miR-26a* | 1,00 | *miR-26a* | 0,36 | *miR-25* | 0,46 | 0,15 | *miR-20a* | 0,18 |
|  | *U6* | 0,18 | *miR-191* | 0,16 | *miR-20a* | 1,68 | *miR-20a* | 0,36 | *U6* | 0,47 | 0,11 | *miR-26a* | 0,23 |
|  | *miR-26a* | 0,19 | *miR-20a* | 0,20 | *miR-191* | 3,22 | *miR-191* | 0,51 | *miR-20a* | 0,47 | 0,13 | *U6* | 0,27 |
|  | *miR-191* | 0,21 | *U6* | 0,23 | *U6* | 4,23 | *U6* | 0,59 | *miR-191* | 0,93 | 0,24 | *miR-191* | 0,28 |
|  | *miR-25* | 0,32 | *miR-25* | 0,28 | *miR-25* | 4,40 | *miR-25* | 0,61 | *miR-26a* | 1,01 | 0,27 | *miR-25* | 0,33 |

All, all samples; CY, cystic; NC, non-cystic; HT, haploinsuficient; WT, wild-type; SC, severe cystic phenotype; CO, severe cystic phenotype controls.

*Best reference genes considering the intra- and intergroup variations.

**Competing Interests:** The authors declare no competing interests.
